# Differences in Player Position Running Velocity at Lactate Thresholds Among Male Professional German Soccer Players

**DOI:** 10.3389/fphys.2019.00886

**Published:** 2019-07-09

**Authors:** René Schwesig, Stephan Schulze, Lars Reinhardt, Kevin G. Laudner, Karl-Stefan Delank, Souhail Hermassi

**Affiliations:** ^1^Department of Orthopedic and Trauma Surgery, Martin-Luther-University of Halle-Wittenberg, Halle (Saale), Germany; ^2^Department of Health Sciences, University of Colorado at Colorado Springs, Colorado Springs, CO, United States; ^3^Sport Science Program, College of Arts and Sciences, Qatar University, Doha, Qatar

**Keywords:** performance diagnostic, endurance, position-specific, treadmill test, team sport, professional athletes

## Abstract

This study investigated the differences in running velocities at specific lactate thresholds among male German soccer players. One hundred fifty-two professional (3rd league: *n* = 78; 4th league: *n* = 74) male soccer players (mean ± SD; age: 24.7 ± 4.37 years, body mass: 80.8 ± 7.33 kg, body height: 1.83 ± 0.06 m) volunteered for the investigation. Players were categorized as goalkeepers, central defenders, central midfielders, wings and forward. Players completed a treadmill test, at incremental speeds, to determine running velocity at different blood lactate concentrations (v2 = 2 mmol/l; v4 = 4 mmol/l; and v6 = 6 mmol/l). In addition, the largest difference between positions for running velocity was found at the lactate threshold v2 (*p* = 0.005). The running data revealed that only goalkeepers had significantly lower velocities at the lactate thresholds compared to outfield players. The central midfielders showed the highest average performance level at the lactate thresholds (v2: 12.5 ± 1.20 km/h; v4: 15.2 ± 1.14 km/h; and v6: 16.6 ± 1.14 km/h). In conclusion, this study provides soccer and position-specific reference data for the running performance of male professional German soccer players to evaluate the endurance performance in a standardized way. In this context, future research should extend the database for the first and second leagues. Further research assessing running performance during competition matches over the entire season is required to validate the endurance test performance data.

## Introduction

Soccer is one of the most widely played and complex sports in the world, where players need technical, tactical, and physical skills to succeed. Analyses of physical performance during short periods in match-play also show that players transiently perform substantially higher amounts of high-intensity running than the game average (Arnason et al., 2004; Faude et al., 2012; Schwesig et al., 2016).

Soccer is classified as a high intensity intermittent team sport (Di Salvo et al., 2009; Krustrup et al., 2009) with high demands on aerobic endurance, strength and the strength derivatives of speed and power. Elite soccer matches are characterized by increased work (e.g., the average oxygen uptake on international level ranges from 55 to 68 ml.kg^–1^.min^–1^) and increased movement velocity (Hoff, 2005). During competitive soccer match play, elite players cover about 10,000–11,000 m at an average intensity close to the anaerobic threshold, being 80–90% of maximal heart frequency (Mohr et al., 2003; Stølen et al., 2005) or 70–80% of maximal oxygen uptake (VO_2_max) (McMillan et al., 2005b). Di Salvo et al. (2007) examined the physical demands from Spanish first league soccer players in relation to their playing position using a computerized match analysis system. Wide (11,990 m) and central midfielders (12,027 m) showed the greatest total distance. While wide midfielders covered the highest distances in the high intensity speed zones (19.1–23.0 km/h: 738 m; >23.0 km/h: 446 m), central midfielders were the players with the highest volume in the middle intensity zones (11.1–14.0 km/h: 1965 m; 14.1–19.0 km/h: 2,116 m). In line with Mohr et al. (2003), central defenders covered the shortest total distance (10,627 m) and distance by high speed running (19.1–23.0 km/h: 397 m; >23.0 km/h: 215 m). They also investigated half time related differences in sprinting distance during competitive matches. The authors reported that forward (19 ± 5%) showed a greater (*p* < 0.05) decline than defenders (11 ± 6%) and midfield players (8 ± 4%). Furthermore, it has been observed that soccer players reach peak running speeds close to 32 km/h during match-play (Rampinini et al., 2007a, b). This quality of sprinting speed depends on several factors including the level of practice and the players’ age (Maffulli et al., 2002; Al Haddad et al., 2015). Indeed, it has been shown that elite players are faster during the first 10 m of a 30 m sprint test than amateurs (Maffulli et al., 2002).

A number of field and laboratory tests are currently used in elite soccer in order to evaluate training status of the players, to predict match performance, and to determine the effect of training (Svensson and Drust, 2005). Various incremental exercise tests and intermittent shuttle run tests are currently used to evaluate training status and training adaptations, as well as to predict running performance during matches in soccer players (Krustrup et al., 2005; Svensson and Drust, 2005). Incremental exercise tests are capable of providing input into aerobic endurance performance parameters (Joyner and Coyle, 2008; O’Brien et al., 2008; Faude et al., 2009; Midgley et al., 2009). In addition, heart rate and lactate thresholds are commonly used as a sensitive indicator of changes in training status in soccer players (Ziogas et al., 2011; de Lucas et al., 2014; Cerda-Kohler et al., 2016; Pallarés et al., 2016).

Altmann et al. (2018) investigated the relationship between test and match performance parameters using data of outfield players (*n* = 28) from the Second German league. This investigation provided valuable information regarding endurance performance diagnostics and match performance data. Comparable with Rampinini et al. (2007a, b), the authors captured maximal running speeds during official matches up to 31.0 km/h. The average total distance was 10,900 m, which was slightly lower than the values reported by Di Salvo et al. (2007); 11,393 m and clearly higher than described by Stølen et al. (2005); 10,000 m. The running speed at the 4 mmol/l lactate threshold (incremental treadmill test) was 15.1 km/h (Altmann et al., 2018). Unfortunately, the authors only investigated 28 soccer players and did not differentiate between playing positions. From a practical point of view, the results of Foehrenbach et al. (1986) are frequently used to judge the running performance of soccer players (McMillan et al., 2005a; Kalapotharakos et al., 2011; Hoppe et al., 2013; Baumgart et al., 2014). A running speed of 4 m/s at the 4 mmol/l lactate threshold is often used by (athletic) coaches as a cut-off point (Foehrenbach et al., 1986). The aim of this study was to assess differences in endurance by playing position measured on a treadmill among male German soccer players. The authors hypothesized that running velocity at various lactate thresholds would be significantly different by playing position in male soccer players.

## Materials and Methods

### Participants

The test data were collected as part of performance diagnostic measures in the period between 2015 and 2018 and resulted from tests with teams from the 3rd and 4th German soccer leagues. A total of 304 data sets were available for the evaluation (3rd league: *n* = 151; 4th league; *n* = 153). To avoid correlated observations, the median from different tests from the same player for every parameter was calculated. After this calculation, 152 professional (3rd league: *n* = 78; 4th league: *n* = 74) male soccer players (mean ± SD; age: 24.7 ± 4.37 years, body mass: 80.8 ± 7.33 kg, body height: 1.83 ± 0.06 m) were included in the statistical analysis. All players were healthy, symptom free, older than 18.0 years and completed the tests following a day of rest. Participation in the investigation was voluntary. All players were informed that they could withdraw from the project at any time without penalty. At the time of the test, the player had to be a member of the soccer team and have a professional contract. None of the players reported any current or ongoing neuromuscular diseases or musculoskeletal injuries specific to the ankle, knee, or hip joints, and none were taking any dietary or performance supplements that might be expected to affect performance during the treadmill test.

To provide an in-depth analysis of team soccer, results were analyzed for the entire group and according to individual playing positions. All the players were fully informed of all experimental procedures and risks before giving their written informed consent to participate. The study was approved by the Ethics Committee of the Martin Luther University of Halle-Wittenberg (Reference Number: 2013-13), conformed to the Declaration of Helsinki (Millum et al., 2013) and met the ethical standards in the Sport and Exercise Science Research Centre (Harriss et al., 2017).

### Experimental Design

This study examined if differences in running velocity at specific lactate thresholds exist between male soccer players of the third league (*n* = 151) and fourth league (*n* = 153) and by playing position. Between leagues, no significant performance differences (v2: *p* = 0.410; v4: *p* = 0.679; and v6: *p* = 0.996) were found. Therefore, comparisons were only made between the following playing positions: goalkeepers (GK), central defenders (CD), central midfielders (CM), wings (WI), and forward (FW).

The position classification for this study was somewhat different than that provided by Bush et al. (2015) and Suarez-Arrones et al. (2015): goalkeeper, central defender, fullbacks, wide midfielder, central midfielder, and attackers. In contrast, in the present study wing players were assigned to tactical role and tasks (wide defender and wide midfielder). The classification of the current study based on own match performance data from the German 3rd and 4th league (*n* = 55; matches of three successive seasons, on average 9 matches per soccer player). These data show, that the running requirements of the wing players (defensive and offensive) are very similar to each other (Rampinini et al., 2007b). The subjects were carefully familiarized with the testing protocol, as they had been previously tested on several occasions in season for training prescription purposes. All the players within a given team were assessed on the same day, and the tests were performed in the same order.

All tests were conducted at the start (May and June 2015–2018) of pre-seasonal training. To reduce the influence of uncontrolled variables, all participants were instructed to maintain their typical lifestyle and diet habits before and during the study. Subjects were told not to exercise on the day ahead of a test and to consume their last (caffeine-free) meal at least 3 h before the scheduled testing. Additionally, they drank at least 0.5 l of pure water during the last hour before testing. Regular sleep ahead of the protocol was also requested. During all performance-based testing, athletes were instructed to perform at their maximum ability.

### Anthropometry Measurement

Body height was measured using a stadiometer (Bodymeter 206, SECA^®^, Hamburg, 164 Germany to 0.1 cm) and body mass on an electrical scale (InBody120, model 165 BPM040S12FXX, Biospace, Inc., Seoul, South Korea, to 0.1 kg).

### Heart Rate Measurement

Heart rate monitors (Polar Team Pro; Polar Electro Oy, Kempele, Finland) were fitted to the players 15 min before the start of the treadmill test. Heart rates were monitored using short-range telemetry with a 1 s recording interval, before, during and after the treadmill test. Athletes were asked to sit in a comfortable position for a duration of at least 10 min without speaking. Heart rate was recorded at the end of each running speed level. The minimum heart rate using a 10 s interval for calculation of the resting heart rate was determined before the treadmill test. The measuring of heart rate was only necessary to monitor the test and calculate heart rate ranges for endurance training.

### Lactate Measurement

The lactate concentration was measured in hemolyzed whole blood using an enzymatic lactate analyser (Super GL easy; Dr. Müller Gerätebau GmbH, Freital, Germany) to determine three running levels. These three levels consisted of: v2 = 2 mmol/l lactate threshold, v4 = 4 mmol/l lactate threshold, and v6 = 4 mmol/l lactate threshold. Blood samples (10 μl capillary blood) were collected from an athlete‘s ear lobe. Afterward test data was analyzed (Winlactat, Mesics, Münster, Germany) to determine the running velocity at the v2, v4, and v6. Mathematical modeling of the lactate-velocity function was determined by either an exponential or a polynomial function, depending on the best fitting results (highest explained variance of the regression function).

### Treadmill Test

Athletes completed a treadmill test at incremental speeds, either in laboratory or gym, to determine running velocity at specific lactate threshold levels (laboratory: quasar, h/p/cosmos, Traunstein, Germany; gym: excite run 1000, Technogym, Neu-Ilsenburg, Germany/260G, pulse fitness, Congleton, United Kingdom). All testing rooms were air conditioned to control the environmental conditions (temperature: 20–22°C; humidity: 40–60%). The test started at a speed level of 7.2 km/h, and an increment of 1.8 km/h every 3 min. The slope was set at 0%. Prior to testing, all subjects completed a 2-min treadmill running warm-up at a speed of 7.2 km/h to adapt to running on the treadmill. The duration of the break between the different speeds was 45 s. During this time the players stepped off the treadmill while their lactate concentration was measured.

### Statistical Analysis

Prior to descriptive and inference statistical analyses, the data was checked for multiple player entries. If there was more than one data set per player, the mean (even number of tests) or the median (odd number of tests) of the test results was calculated and included into the analysis. Results were analyzed for the entire group and for individual playing positions (wings, central defenders, central midfielders, forward and goalkeepers). Descriptive statistics (mean, median, standard deviation, and percentile 10, 25, 50, 75, and 90) were ascertained for all parameters.

Prior to inference statistical analyses, all variables were tested for normal distribution (Kolmogorov–Smirnov-Test) and the assumption of variance homogeneity (Levene-Test for equality of variances). Mean differences between positions were tested using a one-factorial univariate general linear model. Bonferroni tests were used for pairwise analysis to analyze differences at running velocities lactate thresholds between the different playing positions.

Differences between means were considered as statistically significant if *p*-Values were <0.05. To estimate practical relevance and to quantify the performance differences between playing positions, effect sizes (partial eta squared, **$\eta_{p}^{2}$**; Richardson, 2011; *d*; Hartmann et al., 1992) were calculated for the ANOVA main effects (**$\eta_{p}^{2}$**) and the mean differences divided by the pooled standard deviations (SD).

To evaluate effect sizes, *d* or **$\eta_{p}^{2}$** were classified with *d* ≥ 0.2, *d* ≥ 0.5, *d* ≥ 0.8, or **$\eta_{p}^{2}$** ≥ 0.01, **$\eta_{p}^{2}$** ≥ 0.06, **$\eta_{p}^{2}$** ≥ 0.14 indicating small, medium or large effects, respectively (Cohen, 1988).

All statistical analyses were performed using SPSS version 25.0 for Windows (SPSS Inc., Chicago, IL, United States).

## Results

### Normal Distribution and Variance Homogeneity

Only the variable body mass (*p* = 0.039) and BMI (*p* = 0.039) did not have a normal distribution. Regarding variance homogeneity, all *p*-Values were higher than 0.082 (age) indicating that the variances of all variables in the different samples (playing positions) were not significantly different.

### Anthropometric Data

The descriptive statistical analysis (Table 1) displayed differences between positions for body height [mean range: 1.79 m (WI) to 1.90 m (GK)] and body mass [mean range: 76.1 m (WI) to 86.3 m (GK)]. Wings showed the lowest average BMI (23.7 ± 1.41 kg/m^2^) and forward the highest average values (24.5 ± 1.07 kg/m^2^) (Table 1).

**TABLE 1:** Demographic and anthropometric characteristics of soccer players (*n* = 152) in relation to playing positions (Mean ± SD).

	**GK (*n* = 11)**	**CD (*n* = 25)**	**CM (*n* = 37)**	**WI (*n* = 55)**	**FW (*n* = 24)**	**Total (*n* = 152)**
Age (years)	24.2 ± 5.22	25.3 ± 4.86	25.3 ± 4.40	23.7 ± 3.61	25.6 ± 4.85	24.7 ± 4.37
Body height (m)	1.90 ± 0.03	1.87 ± 0.04	1.81 ± 0.05	1.79 ± 0.06	1.87 ± 0.05	1.83 ± 0.06
Body mass (kg)	86.3 ± 5.08	84.8 ± 6.33	80.2 ± 6.65	76.1 ± 6.06	85.4 ± 6.03	80.8 ± 7.33
BMI (kg⋅m^–2^)	23.9 ± 1.36	24.2 ± 1.47	24.4 ± 1.64	23.7 ± 1.41	24.5 ± 1.07	24.1 ± 1.46
Resting heart rate (min^–1^)	66 ± 8.10	61 ± 8.21	64 ± 9.73	62 ± 8.43	65 ± 6.48	63 ± 8.49
Resting lactate (mmol/l)	1.16 ± 0.27	1.06 ± 0.31	1.01 ± 0.27	1.06 ± 0.30	0.98 ± 0.27	1.04 ± 0.29

*GK, goalkeepers; CD, central defenders; CM, central midfielders; WI, wings; FW, forward; SD, standard deviation.*

Resting lactate averaged between 0.98 mmol/l (FW) and 1.16 (GK). The differences regarding resting heart rate were similarly low as in lactate (Table 1). In mean, central defenders showed the minimum (61 beat/min^–1^) and goalkeepers displayed the maximum (66 beat/min^–1^).

### Performance Data

**FIGURE 1:**
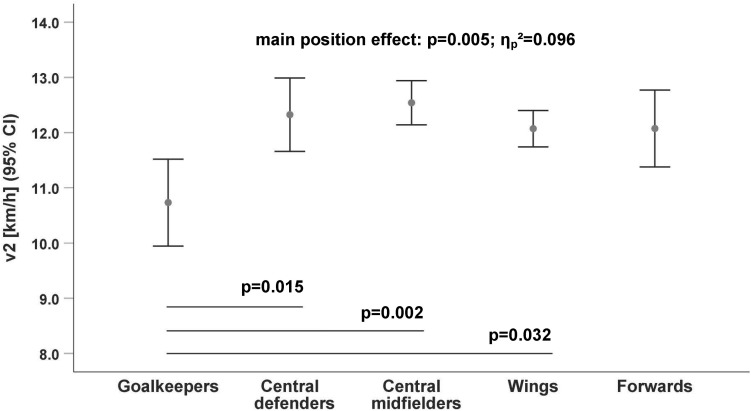
Differences in running velocity by playing position at the 2 mmol/l lactate threshold (v2). CI, Confidence Interval.

**FIGURE 2:**
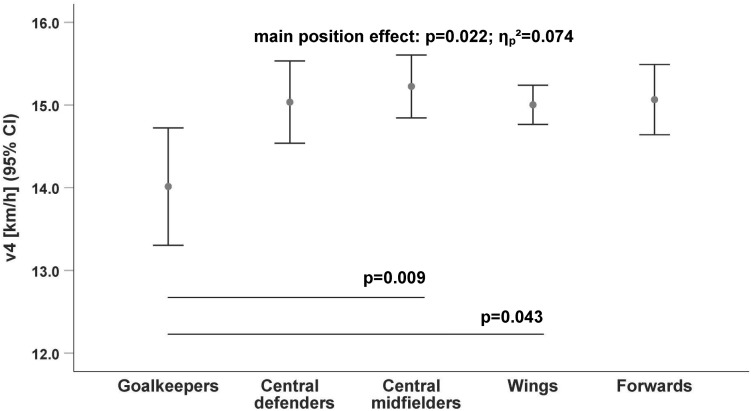
Differences in running velocity by playing position at the 4 mmol/l lactate threshold (v4). Cl, Confidence Interval.

**FIGURE 3:**
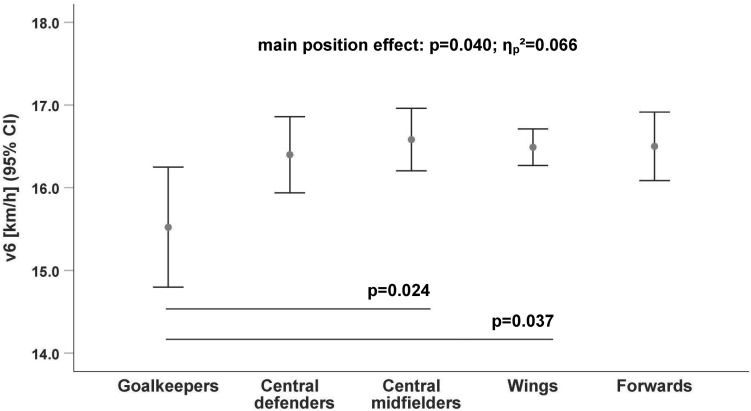
Differences in running velocity by playing position at the 6 mmol/l lactate threshold (v6). Cl, Confidence Interval.

The running data (Table 2) revealed that only goalkeepers have a significantly lower endurance performance compared to outfield players, especially to the central midfielders [v2: *p* = 0.002, *d* = 1.52 (Figure 1); v4: *p* = 0.009, *d* = 1.09 (Figure 2); v6: *p* = 0.024, *d* = 0.99 (Figure 3)].

**TABLE 2:** Percentile data of running velocity (km/h), by playing position, at the different lactate thresholds: 2 mmol/l, 4 mmol/l, and 6 mmol/l.

	**GK (*n* = 11)**	**CD (*n* = 25)**	**CM (*n* = 37)**	**WI (*n* = 55)**	**FW (*n* = 24)**	**Total (*n* = 152)**
**2 mmol/l lactate threshold**						
P10	8.69	9.52	10.9	10.6	9.40	10.4
P25	9.75	11.2	11.6	11.3	11.5	11.3
P50	11.2	**12.7**	12.4	12.1	12.2	12.2
P75	11.4	13.6	13.4	12.9	13.1	13.0
P90	12.5	14.1	14.2	13.8	14.0	14.0
**4 mmol/l lactate threshold**						
P10	12.4	13.2	13.6	13.9	13.7	13.7
P25	12.5	14.4	14.4	14.6	14.3	14.4
P50	14.3	15.1	**15.2**	14.9	15.0	15.0
P75	14.8	16.0	16.0	15.7	15.9	15.7
P90	15.2	16.6	16.6	16.1	16.5	16.4
**6 mmol/l lactate threshold**						
P10	13.9	14.6	15.0	15.4	15.4	15.1
P25	14.1	15.8	15.8	15.9	15.8	15.9
P50	15.8	16.3	**16.5**	**16.5**	16.3	16.5
P75	16.4	17.3	17.3	17.2	17.3	17.2
P90	16.8	17.8	17.9	17.4	18.0	17.7

*Based on the percentile 50, performance maxima are marked in bold. GK, goalkeepers; CD, central defenders; CM, central midfielders; WI, wings; FW, forward.*

The difference between goalkeepers and central midfielders for the lactate threshold v2 (*p* = 0.002; Figure 3) had the largest velocity difference (v_Diff_ = 1.8 km/h; *d* = 1.52) between positions. The endurance performance of the wings was also significantly higher at all lactate thresholds (v2: *p* = 0.032; v4: *p* = 0.043; and v6: *p* = 0.037; Table 3) compared to goalkeepers.

**TABLE 3:** Differences in running velocities [Mean ± SD; (km/h)] at lactate thresholds by playing position (*p* < 0.05).

**Lactate threshold**	**GK (*n* = 11)**	**CD (*n* = 25)**	**CM (*n* = 37)**	**WI (*n* = 55)**	**FW (*n* = 24)**	***p***	**$\eta_{p}^{2}$**
2 mmol/l	10.7 ± 1.17	12.3 ± 1.68	12.5 ± 1.20	12.1 ± 1.19	12.1 ± 1.65	0.005	0.096
GK vs. CD: *p* = 0.015; ***d* = 1.12**							
GK vs. CM: *p* = 0.002; ***d* = 1.52**							
GK vs. WI: *p* = 0.032; ***d* = 1.19**							
4 mmol/l	14.0 ± 1.06	15.0 ± 1.26	15.2 ± 1.14	15.0 ± 0.85	15.1 ± 1.01	0.022	0.074
GK vs. CM: *p* = 0.009; ***d* = 1.09**							
GK vs. WI: *p* = 0.043; ***d* = 1.05**							
6 mmol/l	15.5 ± 1.08	16.4 ± 1.16	16.6 ± 1.14	16.5 ± 0.79	16.5 ± 0.98	0.040	0.066
GK vs. CM: *p* = 0.024; ***d* = 0.99**							
GK vs. WI: *p* = 0.037; ***d* = 1.07**							

*Large effect sizes (*d* ≥ 0.8; $\eta_{p}^{2}$ ≥ 0.14) are marked in bold. GK, goalkeepers; CD, central defenders; CM, central midfielders; WI, wings; FW, forward.*

There were no significant differences among the outfield players (Figures 1–3) whereas the goalkeepers were the players with the lowest performance level at all lactate thresholds (v2: 10.7 ± 1.17 km/h, *p* = 0.005/**$\eta_{p}^{2}$** = 0.096; v4: 14.0 ± 1.06 km/h, *p* = 0.022/**$\eta_{p}^{2}$** = 0.074; and v6: 15.5 ± 1.08 km/h, *p* = 0.040/**$\eta_{p}^{2}$** = 0.066; Table 3). The central midfielders showed the highest average performance level at the lactate thresholds (v2: 12.5 ± 1.20 km/h; v4: 15.2 ± 1.14 km/h; and v6: 16.6 ± 1.14 km/h; Table 3).

## Discussion

The aim of this study was to assess playing position differences in running (endurance) performance among male German soccer players. The findings from this study partially verified the main hypothesis that different playing positions have specific running-related performance requirements among soccer players from the third and fourth German leagues.

In the present study, significant performance differences at different lactate levels (v2, v4, and v6) between playing positions, were only detected between outfield soccer players and goalkeepers. While the goalkeepers always had the lowest running velocity at all lactate thresholds, the central midfielders were the outfield players with the highest average running performance. With increasing lactate levels, the differences between outfield playing positions were decreasing (P50-v2: 0.6 km/h; P50-v4: 0.3 km/h; and P50-v6: 0.2 km/h). This suggests that the endurance training programs, especially the anaerobic workouts, do not have to be so individualized by position (as expected). As expected, soccer players also showed significant differences in anthropometric characteristics, depending on their playing positions.

Although investigations of running performance have been previously conducted by other authors (McMillan et al., 2005a; Kalapotharakos et al., 2011; Ziogas et al., 2011; Hoppe et al., 2013; Baumgart et al., 2014), comparability of these studies is difficult and limited due to differences in methodologies.

Rampinini et al. (2007a) investigated the match performance of professional soccer players using a computerized, semi-automatic video match analysis (speed, distance). Midfielders were the players with the highest average running performance during the match. For example, midfielders covered 4, 13, and 15% more total distance than the fullbacks, forward and center-backs, respectively. The highest values recorded for time spent in the high-speed running zone (19.8–25.2 km/h) were performed by fullbacks and midfielders. These results correspond with the results of the current study, according to which the central midfielders were the players with the highest average running performance.

The quality of lactate threshold data depends partly on the degree of exhaustion, standardization of the subsequent load (Stegmann et al., 1981), or on the kinetics of the test curve (Dickhuth et al., 1991; Cheng et al., 1992). Contrary, evaluating fixed lactate thresholds, especially the 2 mmol/l lactate threshold, can be a disadvantage for fast player types. Some of these players produce high initial lactate values, but they also form higher maximum values at full load, in comparison with players who tend to have more slow twitching muscle fibers (Guner et al., 2005; Kunduracioglu et al., 2007). Longitudinal investigations with soccer players from the 3rd Greek league (Kalapotharakos et al., 2011) showed a clearly lower performance at the 4 mmol/l lactate threshold (12.3–13.7 km/h) with a similar test design (3 min per speed level, with 2 km/h increment). Soccer players of the first three Greek leagues were examined with an almost identical test design to the one used in the current study, but lactate threshold was determined using the Dmax model (Ziogas et al., 2011). Ziogas et al. (2011) reported threshold values (v4) from 3.8 to 3.9 mmol/l, comparable with the results from the current study. The players had an average threshold performance of 13.2 km/h (1st league) to 12.3 km/h (3rd league), which was also strongly different to the findings of the current study (below percentile 10: 13.7 km/h).

Another investigation examined the threshold performance of British youth soccer players (McMillan et al., 2005a). The test design of this study differed significantly from the design of the current investigation (4 min per speed level, with 0.5 km/h increment). During the season v4 speed of the players ranged between 13.6 ± 0.3 and 14.7 ± 0.2 km/h. However, except for preseason performance (13.6 km/h), the other reported values (14.7 km/h) were on a similar level to those of the data from the current study (P50: 15.0 km/h).

Altmann et al. (2018) analyzed the physical capacity of 28 male soccer players (second German league). An incremental treadmill test with a similar test design (start speed: 6 km/h, 3 min per speed level, with 2 km/h increment) was used within a performance testing battery. One of the key parameters for endurance performance was the v4 lactate threshold. The authors reported almost identical results at v4 (average speed: 15.1 km/h, position-independent) for the outfield players compared in the current study (P50: 15.0 km/h). The difference in league (third vs. second) is not a distinguishing criterion.

### Limitations

Due to the support of different teams in different cities, the implementation took place in local fitness studios with the treadmills available there. The test equipment was serviced in the usual maintenance intervals and met the technical specifications specified by the manufacturer. However, some differences in treadmill efficiency could have been present between tests. Therefore, when interpreting the results consideration should be given that differences between treadmills could modify the results obtained during the incremental test. In this context, we controlled all important accuracy factors, that can be influenced by a user [e.g., direct connection to an electrical source (without any extension cabels or power distribution socket), regularly serviced by specialist companies in order to test belt slip and the power supply of the treadmill]. On the other hand, it is indispensable to recruit several teams in different cities to generate a large sample size (*n* = 152). One team cannot provide a sufficient sample size in a defined time interval (e.g., three seasons) in order to calculate reference data. Usually a soccer team consists of 20–25 players. Based on an annual fluctuation rate of 10 players, there is a maximum of 55 players over 3 seasons.

To the best of the authors’ knowledge, there are no uniform guidelines for the running diagnostics of soccer players. Depending on the author collective, the tests for evaluating endurance performance are carried out as field tests on the track or alternatively on the treadmill. The load time per speed level varies between 3 and 5 min (treadmill) or up to 2,400 m per load distance (400 m track) (Foehrenbach et al., 1986; Kalapotharakos et al., 2011; Hoppe et al., 2013). Therefore, the discussion of the different results is difficult, and conclusions should be drawn with great caution.

## Conclusion

In the present study, no significant differences between the outfield players at any lactate level (v2, v4, and v6) were detected. With increasing lactate levels, the differences between outfield playing positions were decreasing. Only differences with the goalkeepers endurance performance were observed, especially when compared to the central midfielders and wings. Over all playing positions, and for a fast judgment of the players in practice, 15.0 km/h (4.2 m/s, median) at the 4 mmol/l lactate threshold should be used. With at least 14.4 km/h (4.0 m/s), the player belongs to the 25% of endurance-weakest athletes and with a running speed of 15.7 km/h (4.4 m/s) to the 25% of the most endurance-capable soccer players (interquartile range: 14.4–15.7 km/h).

The results of this study offer valuable information for soccer players, coaches, and technical staff by providing a soccer-specific classification of endurance performance by playing position. Especially for the pre-season where the endurance training is higher weighted than in other periods of the season, the data can help to evaluate the effectiveness of different endurance programs on several levels (aerobic vs. anaerobic). Further research is needed to clarify the validity of these data and to compare test data with actual soccer match data.

## Data Availability

The datasets for this manuscript are not publicly available because of legal reasons. Requests to access the datasets should be directed to the corresponding author (shermassi@qu.edu.qa).

## Ethics Statement

This study was approved by the Ethics Committee of the Martin-Luther-University of Halle-Wittenberg (Reference Number: 2013-13), conformed to the Declaration of Helsinki (Millum et al., 2013) and met the ethical standards in the Sport and Exercise Science Research Centre (Harriss et al., 2017).

## Author Contributions

RS, SS, and LR carried out the formal analysis and investigated the study. RS, SS, and SH performed the methodology and wrote the final draft of the manuscript. RS and SS carried out the project administration. RS and K-SD supervised the study. KL and SH wrote, reviewed, and edited the final version of the manuscript.

## Conflict of Interest Statement

The authors declare that the research was conducted in the absence of any commercial or financial relationships that could be construed as a potential conflict of interest.
